# Supplementary material for: Chromothripsis during telomere crisis is independent of NHEJ, and consistent with a replicative origin
Source: Genome Res. 2019 May;29(5):737–49. doi: 10.1101/gr.240705.118 (PMC6499312; doi:10.1101/gr.240705.118)
Supplement: Supplemental Material [file supp_gr.240705.118_Supplemental_file_1.zip › contigs/annotated_contigs/DB75/contig.2.DB75_length_363_mean_cov_2.06611570248.docx]

**DB75_length_363_mean_cov_2.06611570248**

```
TTGCATGTAAGTGTCTACGATGAAATTAAGATGTTTATCTTTACAGAGGTACATAA|TTTT|CTAGATGTCAGGTAATTTACCAGAAAG
 >chrX:89601962-89602022 + E=1e-24                       >chrX:89600688-89600995 - E=8e
AGTTGTTTTCTGATCATAACTCTCCTGATGAAAATGGAATCTGGTCCAGCCAGAATGAAGAAACTGACAGACGATGATGATGAAAGCAA
-174
TCCCCAGCTGTCCACATTGCTCATTAGCATAAGACACTCCCACCAGTGCCATCACAGTTTGCAAGTGCCATGGCAACAATTCAGAACTT

ACCACCACTTTCCATAGCAATTACCTAGAAGGTTCCAAACCTTTCCTGGAAAATTCTAAATGACCCGGTCCTCAATTTGCATTGACCAC

CCCTTAATT
```
